# Effects of Physical Exercise on Cognitive Functioning and Wellbeing: Biological and Psychological Benefits

**DOI:** 10.3389/fpsyg.2018.00509

**Published:** 2018-04-27

**Authors:** Laura Mandolesi, Arianna Polverino, Simone Montuori, Francesca Foti, Giampaolo Ferraioli, Pierpaolo Sorrentino, Giuseppe Sorrentino

**Affiliations:** ^1^Department of Movement Sciences and Wellbeing, Parthenope University of Naples, Naples, Italy; ^2^IRCCS Fondazione Santa Lucia, Rome, Italy; ^3^Istituto di Diagnosi e Cura Hermitage Capodimonte, Naples, Italy; ^4^Department of Medical and Surgical Sciences, Magna Graecia University, Catanzaro, Italy; ^5^Department of Science and Technology, Parthenope University of Naples, Naples, Italy; ^6^Department of Engineering, Parthenope University of Naples, Naples, Italy; ^7^Institute of Applied Sciences and Intelligent Systems, CNR, Pozzuoli, Italy

**Keywords:** physical exercise, cognition, wellbeing, brain, epigenetic mechanisms

## Abstract

Much evidence shows that physical exercise (PE) is a strong gene modulator that induces structural and functional changes in the brain, determining enormous benefit on both cognitive functioning and wellbeing. PE is also a protective factor for neurodegeneration. However, it is unclear if such protection is granted through modifications to the biological mechanisms underlying neurodegeneration or through better compensation against attacks. This concise review addresses the biological and psychological positive effects of PE describing the results obtained on brain plasticity and epigenetic mechanisms in animal and human studies, in order to clarify how to maximize the positive effects of PE while avoiding negative consequences, as in the case of exercise addiction.

## Introduction

Many evidences demonstrated that physical exercise (PE) affects brain plasticity, influencing cognition and wellbeing (Weinberg and Gould, 2015; for review see Fernandes et al., 2017). In fact, experimental and clinical studies have reported that PE induces structural and functional changes in the brain, determining enormous biological, and psychological benefits.

In general, when reported PE effects, it is customary to separate the biological aspects from the psychological ones. In fact, most of the studies documented either the effects of PE on the brain (and then on the cognitive functioning) or on the wellbeing (in terms of physical and mental health). In this review, we merge both these aspects as they influence each other. In fact, behaviorally appropriate choices depend upon efficient cognitive functioning. Furthermore, emotional states influence cognitive functions through specific cerebral circuitry involving prefrontal areas and limbic structures (Barbas, 2000).

Before analyzing the benefits of PE, it is necessary to define PE precisely. Indeed, PE is a term often incorrectly used interchangeably with physical activity (PA) that is “any bodily movement produced by skeletal muscles that requires energy expenditure” (World Health Organization, 2010). Then, PA includes any motor behavior such as daily and leisure activities and it is considered a determinant lifestyle for general health status (Burkhalter and Hillman, 2011). Instead, PE is “a sub classification of PA that is planned, structured, repetitive, and has as a final or an intermediate objective the improvement or maintenance of one or more components of physical fitness” (World Health Organization, 2010). Examples of PE are aerobic and anaerobic activity, characterized by a precise frequency, duration and intensity.

In this review, we illustrate the biological and psychological benefits of PE on cognition and wellbeing both in health and diseases, reporting data from both animal and human studies. The biological basis at both molecular and supramolecular level have been largely studied. The other aim of present work is to report the actual evidence on the epigenetic mechanisms that determine or modulate the biological effects of PE on the brain. In fact, while the biologic mechanisms are sufficiently studied both at the molecular and supramolecular levels (see Lista and Sorrentino, 2010), little is known about the epigenetic ones. Finally, the modality with which PE should be practiced to gain such advantages while avoiding negative consequences will be discussed. In Table 1 are reported the inclusion and exclusion criteria for studies discussed in this review.

**Table 1:** Inclusion and exclusion criteria for studies included in this review.

**Inclusion criteria**	**Exclusion criteria**
All studies and review published on indexed journals and indexed in PubMed. Studies related to: -PE effects (biological and psychological effects) - neuroplasticity (structural and functional changes) - correlation between PE and unhealthy behaviors -intensity and modality of PE Published in English Electronically available	Not directly related to PE effects (except in the case of the explanation of neuroplasticity) Not relative to a specific geographic population (for humans)

## Physical exercise, brain, and cognition

Among the biological effects of PE, those linked to “neuroplasticity” are quite important.

Neuroplasticity is an important feature of the nervous system, which can modify itself in response to experience (Bavelier and Neville, 2002). For this reason, PE may be considered as an enhancer environmental factor promoting neuroplasticity.

In animal studies, the structural changes analyzed concern the cellular (neurogenesis, gliogenesis, synaptogenesis, angiogenesis) and molecular (alteration in neurotransmission systems and increasing in some neurotrophic factors) level (Gelfo et al., 2018), while the functional activity has been measured using the levels of performance in behavioral tasks, such as spatial tasks that allow to analyze the different facets of spatial cognitive functions (Mandolesi et al., 2017). In humans, indicators of structural changes correspond for example to brain volumes, measures of white matter integrity or modulation in neurotrophins levels (by correlation with trophic factors plasma levels). Such metrics can be correlated to cognitive performances, defining the functional neural efficiency (Serra et al., 2011). To this regard, it should be emphasized that any morphological change results in a modification of the functional properties of a neural circuit and vice versa any change in neuronal efficiency and functionality is based on morphological modifications (Mandolesi et al., 2017).

Experimental and clinical studies have shown that PE induces important structural and functional changes in brain functioning. In Table 2 are reported the more evident effects induced by PE.

**Table 2:** Structural and functional effects of PE.

**Evidences of PE increasing brain functioning**	
**Animal studies**	**Human studies**
Neurogenesis, synaptogenesis, gliogenesis (hippocampus, neocortex) [1] Angiogenesis (hippocampus, neocortex, cerebellum) [2] Modulation in neurotransmission systems (e.g., serotonin, noradrenalin, acetylcholine) [3] Increased neurotrophic factors (e.g., BDNF, IGF-1) [4] Improvements of spatial memory performances [5] Transgenerational effects of maternal motor exercise [6]	Increased gray matter volume in frontal and hippocampal regions [7] Increased levels of neurotrophic factors (e.g., peripheral BDNF) [8] Increased blood flow [9] Increasing in academic achievement (especially children) [10] Improvements in cognitive abilities (learning and memory, attentional processes and executive processes) [11] Prevention of cognitive decline and reduced risk of developing dementia (especially in the elderly) [12] Modified network topology [13]

[1] van Praag et al., 1999a,b; Brown et al., 2003; Ehninger and Kempermann, 2003; Steiner et al., 2004; Hirase and Shinohara, 2014;

[2] Black et al., 1990; Isaacs et al., 1992; Kleim et al., 2002; Swain et al., 2003; Ekstrand et al., 2008; Gelfo et al., 2018;

[3] Lista and Sorrentino, 2010; Lin and Kuo, 2013;

[4] Vaynman et al., 2004; van Praag, 2009; Lafenetre et al., 2011; Coelho et al., 2013;

[5] van Praag et al., 2005; Nithianantharajah and Hannan, 2006; Langdon and Corbett, 2012; Snigdha et al., 2014;

[6] Akhavan et al., 2008; Aksu et al., 2012; Robinson et al., 2012;

[7] Colcombe et al., 2006; Erickson et al., 2011; Chaddock-Heyman et al., 2014;

[8] Brunoni et al., 2008; Coelho et al., 2013; Hötting et al., 2016;

[9] Weinberg and Gould, 2015; Cabral et al., 2017; Fernandes et al., 2017;

[10] Sibley and Etnier, 2003; Voss et al., 2011; Lees and Hopkins, 2013; Donnelly et al., 2016;

[11] Kramer et al., 1999; Colcombe and Kramer, 2003; Grego et al., 2005; Pereira et al., 2007; Winter et al., 2007; Lista and Sorrentino, 2010; Chieffi et al., 2017; Fernandes et al., 2017;

[12] Colberg et al., 2008; Yaffe et al., 2009; Hötting and Röder, 2013; Niemann et al., 2014; Hollamby et al., 2017; Mandolesi et al., 2017;

[13] *Deeny et al., 2008; Douw et al., 2014; Huang et al., 2016*.

### Animal studies

In animals, motor activity or motor exercise are terms often used instead of PE. The effects of motor exercise are mainly studied in rodents by means of specific training on wheels or by locomotor activity analyses.

Studies on healthy animals have demonstrated that intense motor activity increases neurons and glia cells proliferation rates in the hippocampus and the neocortex (van Praag et al., 1999a,b; Brown et al., 2003; Ehninger and Kempermann, 2003; Steiner et al., 2004; Hirase and Shinohara, 2014) and induces angiogenesis in the neocortex, hippocampus, and cerebellum (Black et al., 1990; Isaacs et al., 1992; Kleim et al., 2002; Swain et al., 2003; Ekstrand et al., 2008; Gelfo et al., 2018). At the molecular level, motor activity causes changes in neurotrasmitters such as serotonin, noradrenalin, and acetylcholine (Lista and Sorrentino, 2010; for a review, see Lin and Kuo, 2013) and induces the release of the brain-derived neurotrophic factor (BDNF Vaynman et al., 2004; Lafenetre et al., 2011) and the insulin-like growth factor-1 (IGF-1; for a review, van Praag, 2009).

Animals performing motor exercise showed improvements in spatial abilities (van Praag et al., 2005; Snigdha et al., 2014) and in other cognitive domains such as executive functions (Langdon and Corbett, 2012), evidencing thus that motor exercise improve cognitive functions.

Similar structural and functional changes were evident even in older animals (Kronenberg et al., 2006) and in animal models of neurodegenerative diseases (Nithianantharajah and Hannan, 2006), suggesting that motor exercise is a potent neuroprotective factor against physiological and pathological aging (Gelfo et al., 2018). In this context, one can use transgenic models to determine exactly when a structural alteration occurs, and then to study when the animals should undergo motor training in order to maximize its effects. To this regard, converging evidence is showing that motor activity should be performed before the development of neurodegeneration in order to exert its protective role (Richter et al., 2008; Lin et al., 2015) such as before the formation of beta amyloid plaques in Alzheimer's disease (Adlard et al., 2005). However, there are some experimental evidences showing that motor exercise performed after neurodegenerative lesions permits to improve spatial abilities, hence being also a potent therapeutic agent (Sim, 2014; Ji et al., 2015).

Interestingly, PE induces modifications that can be passed on to the offspring. In fact, positive maternal experiences can influence the offspring at both behavioral and biochemical levels (see Cutuli et al., 2017, 2018). Preclinical studies also indicated that the effects of maternal exercise during pregnancy can be passed on to offspring (Robinson et al., 2012). However, it is not clear if the possibilities of inheritance are limited to motor exercise alone. To this regard, it has been seen that pregnant rats exposed to motor exercise on wheel-running and treadmill running have offspring with improved spatial memory, and increased hippocampal BDNF level (Akhavan et al., 2008; Aksu et al., 2012). However, further studies are necessary since it remains unclear whether these beneficial effects result from physiological changes to the *in utero* environment and/or from epigenetic modifications to the developing embryo (Short et al., 2017). On the other hand, few studies, conflicting and hard to replicate, do not yet allow to explore the transgenerational effects of paternal motor exercise (Short et al., 2017).

### Human studies

Neuroplasticity phenomena following PE have been evidenced even in humans. A great number of studies demonstrated that in adults, PE determines structural changes such as increased gray matter volume in frontal and hippocampal regions (Colcombe et al., 2006; Erickson et al., 2011) and reduced damage in the gray matter (Chaddock-Heyman et al., 2014).

Moreover, PE facilitates the release of neurotrophic factors such as peripheral BDNF (Hötting et al., 2016), increases blood flow, improves cerebrovascular health and determines benefits on glucose and lipid metabolism carrying “food” to the brain (Mandolesi et al., 2017).

These effects are reflected on cognitive functioning (for a review see Hötting and Röder, 2013). In fact, the results of cross-sectional and epidemiological studies showed that PE enhances cognitive functions in young and older adults (Lista and Sorrentino, 2010; Fernandes et al., 2017), improving memory abilities, efficiency of attentional processes and executive-control processes (Kramer et al., 1999; Colcombe and Kramer, 2003; Grego et al., 2005; Pereira et al., 2007; Winter et al., 2007; Chieffi et al., 2017). Furthermore, structural changes following PE have been related to academic achievement in comparison to sedentary individuals (Lees and Hopkins, 2013; Donnelly et al., 2016). In this line, it has been also showed that children who practice regular aerobic activity performed better on verbal, perceptual and arithmetic test in comparison to sedentary ones of same age (Sibley and Etnier, 2003; Voss et al., 2011).

Numerous studies have demonstrated that PE prevents cognitive decline linked to aging (Yaffe et al., 2009; Hötting and Röder, 2013; Niemann et al., 2014), reduces the risk of developing dementia (Colberg et al., 2008; Mandolesi et al., 2017), the level of deterioration in executive functions (Hollamby et al., 2017) and improves the quality of life (Pedrinolla et al., 2017). Furthermore, positron emission tomography based studies evidenced that PE determines changes in metabolic networks that are related to cognition (Huang et al., 2016).

Recently, studies on magnetoencephalography based (MEG) functional connectivity evidenced that PE influences network topology (Foster, 2015). It is important to underlie that MEG is a much more direct measure of neural activity in comparison to fRMI, with the advantage of combining good spatial and high temporal resolution. In healthy individuals, PE was related to better intermodular integration (Douw et al., 2014) and to improvements in cognitive functions (Huang et al., 2016). Benefits of PE are evidenced even in individuals at risk for AD (Deeny et al., 2008), thus once again suggesting a protective role of PE.

A possible explanation for these ameliorative structural and functional effects could be that PE stimulates blood circulation in the neural circuits involved in cognitive functioning (Erickson et al., 2012). Another interpretation could be found in the concept of “cerebral reserves” (Stern, 2002, 2012) a mechanisms that might explain why, in the face of neurodegenerative changes that are similar in nature and extent, individuals vary considerably in the severity of cognitive aging and clinical dementia (Petrosini et al., 2009). Two types of reserves are recognized: brain reserve and cognitive reserve. The former is based on the protective potential of anatomical features such as brain size, neuronal density and synaptic connectivity, the latter is based on the efficient connectivity among neural circuits (Stern, 2002; Mandolesi et al., 2017).

According to the reserves hypothesis and taking into account the numerous evidences described above, we could advance that PE is an environmental factor that permits to gain reserves.

However, one must underline that if on the one hand PE improves the cognitive functioning, providing reserves to be spent in the case of a brain lesion, on the other hand the modifications of the clinical expression of neurodegeneration delays the diagnosis. It has been seen that patients with higher cognitive reserve take longer to manifest the symptoms of memory loss (Zanetti et al., 2017). It has been hypothesized a neural compensation mechanism that permits to perform complex activities (Stern, 2009). Obviously, these conclusions open important reflections more for the diagnosis of neurodegenerative disease than for the practice of PE.

The effects of PE on cognitive functioning have been shown across the lifespan from childhood to the old age (Hötting and Röder, 2013). In particular, it has been evidenced that cognitive functions that are influenced the most by brain maturation, such as attention or cognitive flexibility, and the cognitive functions that depend the most upon experiences, such as memory, are the most sensitive ones to PE (Hötting and Röder, 2013). Overall, these studies, together with those analyzing the effects of combined environmental factors, suggest that for a positive effect on cognitive function, it is necessary to maintain an “enriched lifestyle” up to middle life. In fact, the exposure to PE together to other many experiences provides a “reserve”-like advantage which supports an enduring preservation of cognitive function in old age (Chang et al., 2010; Loprinzi et al., 2018).

## Physical exercise and wellbeing

There are consistent evidences that PE has many benefits for people of any age, improving psychological wellbeing (Zubala et al., 2017) and quality of life (Penedo and Dahn, 2005; Windle et al., 2010; Table 3).

**Table 3:** Biological and psychological effects of PE (Adapted from Weinberg and Gould, 2015).

**PE effects on psychological wellbeing**	
**Biological effects**	**Psychological benefits**
Increased cerebral blood flow, maximal oxygen consumption and delivery of oxygen to cerebral tissue, reduction in muscle tension, increased serum concentrations of endocannabinoid receptors [1] Cerebral structural changes, increased levels of neurotransmitters (e.g., serotonin, beta-endorphins) [2]	PE decreases: anxiety, depression, dysfunctional and psychotic behaviors, hostility, tension, phobias, headaches [3] PE increases: assertiveness, confidence, emotional stability, cognitive functioning, internal locus of control, positive body image, self-control, sexual satisfaction [4]

[1] Thomas et al., 1989; Dietrich and McDaniel, 2004; Querido and Sheel, 2007; Gomes da Silva et al., 2010; Ferreira-Vieira et al., 2014;

[2] Young, 2007; Korb et al., 2010; Fuss et al., 2015;

[3] Martinsen, 1990; Scully et al., 1998; Craft and Perna, 2004; De Moor et al., 2006; Knapen et al., 2009; Carek et al., 2011; Vatansever-Ozen et al., 2011; DeBoer et al., 2012; Haasova et al., 2013; Mammen and Faulkner, 2013; de Souza Moura et al., 2015; Tiryaki-Sonmez et al., 2015; Weinberg and Gould, 2015; Meyer et al., 2016a,b;

[4] *Marsh and Sonstroem, 1995; Fox, 2000; Berger and Motl, 2001; Landers and Arent, 2001; Urso and Clarkson, 2003; Craft, 2005; Penedo and Dahn, 2005; Raedeke, 2007; Stessman et al., 2009; Bartlett et al., 2011; Biddle et al., 2011; Rodgers et al., 2014; Zamani Sani et al., 2016*.

In children, PE is correlated with high levels of self-efficacy, tasks goal orientation, and perceived competence (Biddle et al., 2011). In youth and adulthood, most studies evidenced that PE is associated with better health outcomes, such as better mood and self-concept (Berger and Motl, 2001; Landers and Arent, 2001; Penedo and Dahn, 2005). In the aging population, PE helps maintaining independence (Stessman et al., 2009), favoring social relations and mental health.

It was now well-accepted that is the interaction between biological and psychological mechanisms linked to PE enhances the wellbeing (Penedo and Dahn, 2005). Biological mechanisms of beneficial effects of PE are mainly related to increasing in cerebral blood flow and in maximal oxygen consumption, to delivery of oxygen to cerebral tissue, to reduction in muscle tension and to increased serum concentrations of endocannabinoid receptors (Thomas et al., 1989; Dietrich and McDaniel, 2004; Querido and Sheel, 2007; Gomes da Silva et al., 2010; Ferreira-Vieira et al., 2014). Moreover, neuroplasticity phenomena such as changes in neurotransmitters are recognized to affect wellbeing. For example, PE increases the levels of serotonin (Young, 2007; Korb et al., 2010) and the levels of beta-endorphins, such as anandamide (Fuss et al., 2015).

Among the psychological hypothesis proposed to explain how PE enhances wellbeing, it has been underlined feeling of control (Weinberg and Gould, 2015), competency and self-efficacy (Craft, 2005; Rodgers et al., 2014), improved self-concept and self-esteem (Marsh and Sonstroem, 1995; Fox, 2000; Zamani Sani et al., 2016), positive social interactions and opportunities for fun and enjoyment (Raedeke, 2007; Bartlett et al., 2011).

Psychological research evidenced that PE can even modulate the personality and the development of Self (Weinberg and Gould, 2015). Moreover, PE has been correlated with hardiness, a personality style that enables a person to withstand or cope with stressful situations (Weinberg and Gould, 2015).

In the following sections, we will focus on correlations among PE and the most common mental illnesses.

### Depression and anxiety

Depression is the most common type of mental illness and will be the second leading cause of disease by 2020 (Farioli-Vecchioli et al., 2018). Similar entity concerns anxiety disorders that are among the most prevalent mental disorders in the world population (Weinberg and Gould, 2015).

Epidemiological studies have consistently reported benefits of PE on reductions in depression (Mammen and Faulkner, 2013) and anxiety (DeBoer et al., 2012). For example, it has been seen that individuals that practice PE regularly are less depressed or anxious than those who do not (De Moor et al., 2006), suggesting the use of exercise as a treatment for these illnesses (Carek et al., 2011).

Most of the research on the relationship between PE and positive changes in mood state has evidenced positive effects, especially as a consequence of aerobic exercise, regardless of the specific type of activity (Knapen et al., 2009), even if the correct intensity of aerobic PE to control and reduce symptoms is debated (de Souza Moura et al., 2015). For example, it has been revealed that after about 16 weeks of an aerobic exercise program, individuals with major depressive disorder (MDD), significantly reduced their depressive symptoms (Craft and Perna, 2004). However, there are evidenced that documented that even anaerobic activity has positive effects on treatment of clinical depression (Martinsen, 1990). For anxiety disorders, it has been evidenced that the positive effects of PE are visible even with short bursts of exercise, independently from the nature of the exercise (Scully et al., 1998).

A physiologic mechanism correlated to the improvement in depressed mood post-exercise PE was identified in modulation of peripheral levels of BDNF (Coelho et al., 2013). In this line, it was suggested recently that the intensity of exercise to improve mood should be prescribed on individual basis and not on the patient's preferred intensity (Meyer et al., 2016a,b). Conversely, physical inactivity correlated to worse depressive symptoms and, then, to lower peripheral levels of BDNF (Brunoni et al., 2008). Post-PE mood improvement might also be due to lower oxidative stress (Thomson et al., 2015). In this contest, it was evidenced that there is an abnormal oxidative stress in individuals with MDD or bipolar disorder (Cataldo et al., 2010; Andreazza et al., 2013) and that PE, particularly in higher intensity, decreases oxidative stress with consequent mood improvement (Urso and Clarkson, 2003).

### Addictive and unhealthy behaviors

PE has been widely evidenced to be an effective tool for treating several addictive and unhealthy behaviors. PE tends to reduce and prevent behaviors such as smoking, alcohol, and gambling, and to regulate the impulse for hunger and satiety (Vatansever-Ozen et al., 2011; Tiryaki-Sonmez et al., 2015). In this context, several studies evidenced substance abusers benefit from regular PE, that also helps increasing healthy behaviors (Giesen et al., 2015). It has been evidenced that regular PE reduces tobacco cravings and cigarette use (Haasova et al., 2013). Although PE has positive effects on psychological wellbeing, in this context it is right underline that in some cases PE could reveal unhealthy behaviors with negative consequence on health (Schwellnus et al., 2016). It is the case of exercise addiction, a dependence on a regular regimen of exercise that is characterized by withdrawal symptoms, after 24–36 h without exercise (Sachs, 1981), such as anxiety, irritability, guilt, muscle twitching, a bloated feeling, and nervousness (Weinberg and Gould, 2015). There is a strong correlation between exercise addiction and eating disorders (Scully et al., 1998) suggesting thus a comorbidity of these disorders and a common biological substrate. In particular, recent studies have shown that these unhealthy behaviors are associated to lower prefrontal cortex volume, activity and oxygenation, with consequent impairment in cognitive functions, such as the inhibitory control with the consequent compulsive behaviors (Asensio et al., 2016; Wang et al., 2016; Pahng et al., 2017). Also, it has been seen that a few days of PE increase oxygenation of prefrontal cortex, improving mental health (Cabral et al., 2017).

## Epigenetic mechanisms

Biological and psychological effects of PE could be partly explained through epigenetic mechanisms. The term “epigenetics,” coined by Waddington (1939), is based on a conceptual model designed to account for how genes might interact with their environment to produce the phenotype (Waddington, 1939; Fernandes et al., 2017).

In particular, epigenetics is referred to all those mechanisms, including functional modifications of the genome such as DNA methylation, post-translational histone modifications (i.e., acetylation and methylation) and microRNA expression (Deibel et al., 2015; Grazioli et al., 2017), which tend to regulate gene expression, modeling the chromatin structure but maintaining the nucleotide sequence of DNA unchanged.

The current literature clearly demonstrates that these mechanisms are strongly influenced by different biological and environmental factors, such as PE (Grazioli et al., 2017), which determine the nature and the mode of epigenetic mechanisms activation.

Epigenetics plays an essential role in neural reorganization, including those that govern the brain plasticity (Deibel et al., 2015). For example, a growing body of evidence indicates that regulates neuroplasticity and memory processes (Ieraci et al., 2015).

Several animal studies reveal how motor activity is able to improve cognitive performances acting on epigenetic mechanisms and influencing the expression of those genes involved in neuroplasticity (Fernandes et al., 2017). The main molecular processes that underlie the epigenetic mechanisms are the following: through DNA methylation, histone modifications and microRNA expression (Fernandes et al., 2017).

DNA methylation is a chemical covalent modification on the cytosine of the double stranded DNA molecule. It has been recognized that DNA methylation plays a key role in long-term memory (Deibel et al., 2015; Kim and Kaang, 2017). In particular, mechanisms related to DNA methylation relieve the repressive effects of memory-suppressor genes to favor the expression of plasticity-promoting and memory consolidation genes. Several evidences showed that PE is able to coordinate the action of the genes involved in synaptic plasticity that regulate memory consolidation (Molteni et al., 2002; Ding et al., 2006).

Histone modifications are post-translational chemical changes in histone proteins. They include histone methylation/demethylation, acetylation/deacetylation, and phosphorylation, all due to the activity of specific enzymes, which modify the chromatin structure, thereby regulating gene expression. It has been demonstrated that histone acetylation is a requisite for long-term memory (LTM) (Barrett and Wood, 2008; Fernandes et al., 2017). In animals, motor activity increases these genetic mechanisms in the hippocampus and the frontal cortex, improving memory performances in behavioral tasks (Cechinel et al., 2016). Recently, following 4 weeks of motor exercise, it has been evidenced an increasing of the activity of enzymes involved in histone acetylation/deacetylation, the epigenetic mechanisms that determine an enhancing in the expression of BDNF (Maejima et al., 2018).

MicroRNAs (miRNAs) are small, single stranded RNA molecules able to inhibit the expression of target genes. They are widely expressed in the brain, participating in epigenetic mechanisms and acting as regulators of numerous biological processes in the brain, ranging from cell proliferation, differentiation, apoptosis, synaptic plasticity, and memory consolidation (Saab and Mansuy, 2014). Recent evidences demonstrate that PE can mitigate the harmful effects of traumatic brain injury and aging on cognitive function by regulating the hippocampal expression of miR21 (Hu et al., 2015) and miR-34a (Kou et al., 2017). Furthermore, PE contributes to attenuate the effects of stress-related increase in miR-124, involved in neurogenesis and memory formation (Pan-Vazquez et al., 2015).

## What kind of physical exercise?

Sport psychology has suggested that the success or failure of PE programs depends on several factors such as the intensity, frequency, duration of the exercise, and whether the PE is done in group or alone (Weinberg and Gould, 2015). These aspects are important in terms of maintenance of PE practice and in order to gain benefits for brain and behavior, and they are affected by individual characteristics. Although such aspects have to be taken into account when training is proposed, scientific reports have evidenced different effects on cognitive functioning and wellbeing if PE is performed in aerobic or anaerobic modality.

Aerobic exercise allows the resynthesis of adenosine—triphosphate (ATP) by aerobic mechanisms, adjusting intensity (from low to high intensity), duration (usually long), and oxygen availability. The intensity depends on the cardiorespiratory effort with respect to the maximum heart rate (HRmax) or the maximum oxygen consumption (Vo2max), which determines an increase in oxygen consumption with respect to the rest condition. Examples of aerobic PE are jogging, running, cycling, and swimming.

On the contrary, anaerobic exercise has high intensity, short duration and unavailability of oxygen, determining the depletion of the muscles' ATP and/or phosphocreatine (PCr) reserves, shifting the production of ATP, to anaerobic energy mechanisms, lactacid or alactacid. Examples of anaerobic exercises are weight lifting or sprint in 100 m.

Robust literature demonstrated that chronic aerobic exercise is associated with potent structural and functional neuroplastic changes, with an improvement in cognitive functions (Colcombe et al., 2006; Hillman et al., 2008; Erickson et al., 2009; Mandolesi et al., 2017) and increased feeling of general wellbeing (Berger and Tobar, 2011; Biddle et al., 2011) (Table 4).

**Table 4:** Effects of physical aerobic exercise on cognitive functioning and wellbeing.

**Physical aerobic exercise**	
**Chronic aerobic exercise** Several months moderate/high intensity (%VO_2_max 40 to ≥60) moderate duration (16–45 min)	**Acute aerobic exercise** Single bout of PE moderate/high intensity (%VO_2_max 40 to ≥60) with different protocols
Increasing in neuroplasticity phenomena Improvement in cognitive function (especially memory and executive functions) Counteracts neurodegeneration (to prevent, delay or treat cognitive decline) General wellbeing Decreasing anxiety and depression	Enhances affective, mood, and emotional states in healthy individuals Improves the mood and wellbeing in MDD individuals Improvement in cognitive function
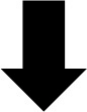	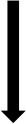
**major positive effects** (for references see Tables 2, 3)	**Small and/or debated positive effects** (Tomporowski, 2003; Bartholomew et al., 2005; Lambourne and Tomporowski, 2010; Chang et al., 2011, 2012; Ludyga et al., 2016; Basso and Suzuki, 2017)

Recently, growing evidence showed that acute aerobic exercise, defined as a single bout of exercise, relates to improved cognitive functions, especially prefrontal cortex-dependent cognition (Tomporowski, 2003; Lambourne and Tomporowski, 2010; Chang et al., 2011; Ludyga et al., 2016; Basso and Suzuki, 2017). However, the effects of a single session of exercise on cognitive functioning are generally small (Chang et al., 2012). In this line, it was evidenced that even a single bout of moderate-intensity aerobic exercise enhances, mood and emotional states and improves the wellbeing in MDD individuals (Bartholomew et al., 2005; Basso and Suzuki, 2017) (Table 4).

Beside frequency and duration over time, even the intensity is a parameter to be considered when evaluating the PE effects. It has been showed that moderate intensity exercise is related to increased performance in working memory and cognitive flexibility, whereas high-intensity exercise improves the speed of information processing (Chang and Etnier, 2009). In this context, it has been reported that peripheral BDNF was significantly increased after high intensity exercise, but not after low-intensity exercise (Hötting et al., 2016). In fact, it is evidenced that high-intensity exercise provides greater benefit to cognitive functions than low-intensity exercise in the elderly (Brown et al., 2012).

With regard to the psychological beneficial effects related to PE, research has evidenced that major benefits in reduction of anxiety and depression are determined by longer training program (several months), as compared to shorter ones (some days) for training session lasting over 30 min. Moreover, anxiety and depression reduction after aerobic exercise may be achieved with exercise intensity between 30 and 70% of maximal heart rate (Weinberg and Gould, 2015). To achieve positive mood changes, an important role is played even by anaerobic activity, such as yoga, or in all PEs in which there is rhythmic abdominal breathing, enjoyment, rhythmic, and repetitive movements and relative absence of interpersonal competition (Berger and Motl, 2001).

## Conclusion

PE determines positive biological and psychological effects that affect the brain and the cognitive functioning and promote a condition of wellbeing. PE plays an important role in counteract normal and pathological aging. Recent evidences have shown that PE triggers potent neuroplastic phenomena, partly mediated by epigenetic mechanisms. In fact, PE cause profound alterations in gene expression and its protein products in the form of epigenomic manifestations (Fernandes et al., 2017).

A growing body of literature indicates that both chronic and aerobic PE can achieve similar benefits.

These results should lead to reflect on beneficial effects of PE and to promote its use as a modifiable factor for prevention, to improve cognitive abilities and to enhance mood.

Despite all these positive effects, it must be underlined that PE should be tailored to the individual. In fact, even PE, when excessive, can have a dark side, when PE becomes compulsive and facilitates addictive behaviors.

## Author contributions

LM, AP, SM, FF, GF, PS, and GS: designed the review; LM and GS: wrote the paper. All authors read, revised, and approved the final manuscript.

### Conflict of interest statement

The authors declare that the research was conducted in the absence of any commercial or financial relationships that could be construed as a potential conflict of interest.
